# Microglial TonEBP mediates LPS-induced inflammation and memory loss as transcriptional cofactor for NF-κB and AP-1

**DOI:** 10.1186/s12974-020-02007-9

**Published:** 2020-12-08

**Authors:** Gyu Won Jeong, Hwan Hee Lee, Whaseon Lee-Kwon, Hyug Moo Kwon

**Affiliations:** School of Life Sciences, Ulsan National Institute of Science and Technology, Ulsan, 44919 Republic of Korea

**Keywords:** Microglial activation, Neuronal cell death, TonEBP

## Abstract

**Background:**

Microglia are brain-resident myeloid cells involved in the innate immune response and a variety of neurodegenerative diseases. In macrophages, TonEBP is a transcriptional cofactor of NF-κB which stimulates the transcription of pro-inflammatory genes in response to LPS. Here, we examined the role of microglial TonEBP.

**Methods:**

We used microglial cell line, BV2 cells. TonEBP was knocked down using lentiviral transduction of shRNA. In animals, TonEBP was deleted from myeloid cells using a line of mouse with floxed TonEBP. Cerulenin was used to block the NF-κB cofactor function of TonEBP.

**Results:**

TonEBP deficiency blocked the LPS-induced expression of pro-inflammatory cytokines and enzymes in association with decreased activity of NF-κB in BV2 cells. We found that there was also a decreased activity of AP-1 and that TonEBP was a transcriptional cofactor of AP-1 as well as NF-κB. Interestingly, we found that myeloid-specific TonEBP deletion blocked the LPS-induced microglia activation and subsequent neuronal cell death and memory loss. Cerulenin disrupted the assembly of the TonEBP/NF-κB/AP-1/p300 complex and suppressed the LPS-induced microglial activation and the neuronal damages in animals.

**Conclusions:**

TonEBP is a key mediator of microglial activation and neuroinflammation relevant to neuronal damage. Cerulenin is an effective blocker of the TonEBP actions.

**Supplementary Information:**

The online version contains supplementary material available at 10.1186/s12974-020-02007-9.

## Introduction

Neuroinflammation is inflammation in the brain or spinal cord in response to a variety of insults or cues including infection, brain injury, or aging. Like other forms of inflammation, neuroinflammation is mediated by the production of cytokines, chemokines, reactive oxygen species (ROS), and secondary messengers [1]. These inflammatory factors are produced by various cell types in the central nervous system (CNS) such as microglia, astrocytes, endothelial cells, other glial cells, and peripherally derived immune cells. Neuroinflammation can be protective or pathogenic depending on the context and type of molecules involved [1].

Microglia are resident macrophages of the CNS. During embryonic development, primitive macrophages generated in the yolk sac give rise to embryonic microglia [2]. After birth, bone marrow-derived macrophages cross blood-brain barrier and supplement the microglial population. Microglia serve two major functions [3]. Microglia contribute to maintenance of CNS homeostasis by controlling neuronal proliferation and differentiation. Microglia also play a critical role in innate immunity in CNS and brain disease. In settings of pathogenesis, inflammatory stimuli can prime microglial cell leading to a constant production of inflammatory cytokines and chemokines which, in turn, maintain activation of the primed cells [4]. These cycles finally lead to neuronal loss and neurodegeneration via inflammatory pathways or activation of A1 astrocytes [5, 6].

Tonicity-responsive enhancer binding protein (TonEBP), also known as nuclear factor of activated T cells 5 (NFAT5), belongs to the Rel family of transcription factors, which includes nuclear factor-κB (NF-κB) and NFAT 1–4 [7, 8]. TonEBP is involved in a variety of physiological and pathological processes by controlling transcription of different sets of genes through distinct mechanisms [9]. For example, in the renal medulla, TonEBP stimulates transcription of aquaporin 2 as a *cis*-acting transcriptional enhancer [10]. In macrophages, TonEBP regulates transcription of pro-inflammatory genes such as tumor necrosis factor-α (TNF-α) and cyclooxygenase-2 (COX-2) as a transcription cofactor of NF-κB independent of DNA binding [11]. As such, TonEBP is a key regulator of systemic inflammation.

In the brain, TonEBP expression is limited to neurons without detectable expression in glial cells or other non-neuronal cells [12]. We recently reported that TonEBP expression is specifically elicited in microglia in response to direct injection of lipopolysaccharide (LPS) [13]. Here, we investigated the role of TonEBP in microglia. Our data show that TonEBP is a key mediator of microglial activation and neuroinflammation relevant to neuronal damage.

## Methods

### Cells in culture

BV2 cells, a mouse microglial cell line (cat. no. CRL-2467, ATCC) [14], was cultured in Dulbecco’s Modified Eagle’s Medium (DMEM) containing 5% fetal bovine serum (FBS, Thermo Fisher Scientific, Inc., MA, USA) and penicillin/streptomycin (100 U/ml and 100 μg/ml; GE Healthcare Life Sciences, UT, USA). For HT22 cells, a mouse hippocampus neuronal cell line (SCC129) [15] and mouse embryonic fibroblasts (MEFs) cells [11] DMEM containing 10% FBS was used. Cells were maintained at 37 °C in an incubator with 5% CO_2_. BV2 cells were transfected with TonEBP shRNA-harboring lentiviral particles (Santa Cruz Biotechnology) in the presence of polybrene (5 μg/ml). Stable cells were selected in the presence of puromycin (10 μg/ml). Cells were pretreated with cerulenin and BAY 11-7082 (Sigma Aldrich, USA) for 1 h and exposed to lipopolysaccharide (LPS; Sigma Aldrich).

### Immunoblot assay

Cells were washed twice with 1x cold phosphate-buffered saline (PBS) and lysed in RIPA buffer: 0.01 M Tris, pH 7.4, 0.15 M NaCl, 0.001 M EDTA, 0.001 M EGTA, 1% Triton X-100, phosphatase inhibitor cocktail (Sigma Aldrich), and protease inhibitor (Roche, Rotkreuz, Switzerland). The lysates were cleared by centrifugation for 15 min at 17,000 *g*. Protein concentration was measured by BCA assay (Pierce Biotechnology, IL, USA). Equal amounts of protein (30 μg) were separated by SDS-PAGE and immunoblotted with specific primary antibodies overnight at 4 °C. 1:1000 Anti-c-jun (cat. no. 60A8, Cell Signaling Technologies, Berkeley, CA, USA), 1:1000 p65 (cat. no. D14E12, Cell Signaling Technologies), 1:1000 COX-2 (cat. no. 4842S, Cell Signaling Technologies), 1:10000 anti-actin (A5441, Sigma Aldrich, USA), and 1:3000 anti-TonEBP antibody [7] were used. 1:10000 HRP-conjugated mouse or rabbit secondary antibodies were used for detection. Antigen-antibody binding was detected by chemiluminescent detection reagent (GE Healthcare, NJ, USA).

### RNA isolation and quantitative PCR

Total RNA from BV2 cells and mouse hippocampus and cortex was isolated using TRIzol reagent (Invitrogen, CA, USA) according to the manufacturer’s instructions. cDNA was synthesized by M-MLV reverse transcriptase (Promega). After cDNA synthesis, quantitative PCR was performed with SYBR Green I Master Mix and a LightCycler 480 II instrument (Roche) using primers shown in Supplementary Table S1. RNA quantity was normalized to the cyclophilin A mRNA.

### ELISA

TNF-*α* in cell culture media was analyzed by ELISA using a commercial kit (cat. no. MTA00B, R&D Systems, MN, USA).

### MTT assay

1.5 × 10^6^ BV2 cells were seeded in 10 ml of culture medium and grown overnight. The medium was replaced by 5 ml of medium containing 100 ng/ml of LPS, and then, cultured 24 h to obtain conditioned medium. Each conditioned medium was filtered with a 0.45 μ membrane. HT22 cells were seeded on 96-well plates at a density of 1 × 10^4^ cells per well with 0.2 ml culture medium. After overnight culture, the medium was replaced by the microglia conditioned medium. Twenty-four hours later, 20 μl of 50 mg/ml 3-(4,5-dimethylthiazol-2-yl)-2,5-diphenyltetrazolium bromide (MTT) was added. Reduced MTT was measured by absorbance at 490 nm.

### TUNEL assay

HT22 cells were plated on coverslips and grown overnight. The cells were then switched to the microglia-conditioned medium. After 24 h, TUNEL assay was performed using the DeadEnd^TM^ Fluorometric TUNEL System (cat. no. G3250, Promega, WI, USA) following manufacturer’s instructions.

### Luciferase assay

Cells were transfected with either an AP-1 luciferase reporter (3xAP1pGL3, www.addgene.org) or a κB-driven luciferase plasmid [11]. Luciferase activity was measured using the Dual-Luciferase Assay System (cat. no. E1910, Promega) as described [11].

### Immunoprecipitation assay

MEF cells were treated without or with 10 μM cerulenin (Sigma Aldrich) for 1 h followed by a 1 h treatment with 100 ng/ml LPS. Cell lysates were immunoprecipitated overnight at 4 °C using an anti-c-jun antibody (1:50 dilution) (Cat. No. 60A8, Cell Signaling Technologies) as described previously [11].

### Mice

All methods involving live mice were carried out in accordance with approved guidelines. All experimental protocols were approved by the Institutional Animal Care and Use Committee of the Ulsan National Institute of Science and Technology (UNISTACUC-12-15-A). *TonEBP*^*fl/fl*^ *LysM-Cre* and *TonEBP*^*fl/fl*^ mice described previously [11] and C57BL/6 J mice were used. The littermates were kept together in the same cage. *LysM-Cre* mice were purchased from the Jackson Laboratory (cat. No. 004781, Bar Harbor, ME, USA). For experiments, 8-week-old male mice were used.

### Stereotaxic surgery

Eight-week-old male mice were anesthetized by intraperitoneal injection of zoletil (20 mg/kg) and rompun (5 mg/kg). Animals were positioned on a stereotaxic apparatus and received 3 μl LPS (1 μg/μl 1x PBS) or 1x PBS at a 1 μl/min rate into the left ventricle (0.6 mm posterior; 1.2 mm lateral; 1.8 mm ventral from bregma) following the previously reported stereotaxic coordinates [16]. Some animals received administration of 2 mM cerulenin in 3 μl water (6 nmol) or water alone at a rate of 1 μl/min.

### Immunohistochemistry

For immunohistochemistry, mice were anesthetized by injecting a mixture of zoletil and rompun as described above. After perfusion with 1x PBS containing 150 mM NaCl, 60 mM nitrate, and heparin (200u/ml), whole brain was excised and fixed in 8% paraformaldehyde in PBS (pH 7.4) at 4 °C. After 1 day, the fixative solution was replaced with 30% sucrose for an additional 1 day at 4 °C. The fixed brains were cryosectioned at 30 μm and stored in stock solution containing glycerol, ethylene glycol, and 0.2 M phosphate buffer. For immunofluorescence detection, sections were washed twice with 1x PBS for 15 min and permeabilized with 0.2% Triton X-100 in BSA/PBS for 1 h. For immunostaining, sections were incubated with 1:500 anti-Iba-1 antibody (cat. no. 019-19741, Wako, Richmond) or 1:500 anti-NeuN antibody (cat. no. MAB377, Millipore) in 0.5% BSA/PBS overnight at 4 °C. Antibody binding was detected with goat anti-rabbit Alexa Fluor 594 or goat anti-mouse Alexa Fluor 594-conjugated secondary antibodies respectively.

### Behavior tests

#### Passive avoidance test

Passive avoidance test was used to access short-term memory [17]. Passive avoidance apparatus (cat. no. LE872, Panlab) consists of a light and a dark compartment with an automated sliding door, across which mice can pass. All the tests were performed at 1:00 PM which is in resting phase (light period 06:00–18:00). On day 1 of the experiment, mice were positioned in the light compartment and allowed 30 s of exploration. The sliding door was then opened to induce movement to the dark compartment. This process is repeated 3 times for habituation. On day 2 of the experiment, the same protocol was conducted, but entry into the dark compartment was punished with a mild inescapable electrical shock (0.15 mV). On day 3 of the experiment, the procedure was repeated, and the latency time of each mouse was measured, with a maximum latency time of 5 min. Before each trial, to erase any scent cues, the interior of the maze was sprayed with 70% ethanol. All behavioral experiments were conducted over the same time frame.

#### Y-maze tests

Y-maze test was used to measure spatial working memory by spontaneous alteration [18–20]. Each mouse was placed in the same arm of the maze and allowed to move freely for 10 min. The number of different arm choices and the sequence of choices were recorded to assess percent alteration. Prior to each trial, to erase any scent cues, the interior of the maze was sprayed with 70% ethanol.

### Nuclear fractionation

Cell lysates were centrifuged at 500 *g*. The cell pellet was washed by suspension with 1x PBS. Nuclear and Cytoplasmic extraction kit (cat. no. 78833, Pierce) according to manufacturer’s instruction was used for separating the cell nucleus and cytoplasm. Nuclear fraction was confirmed by Lamin B.

### Statistical analysis

Data are expressed as the mean + SD or SEM. Statistical significance was estimated using one-way ANOVA or two-way ANOVA with Tukey’s post hoc test for multiple comparisons where applicable. All statistical analyses were performed using the GraphPad Prism 5.0 software (GraphPad, CA, USA).

## Results

### TonEBP is required for inflammation in BV2 cells

**Fig. 1:**
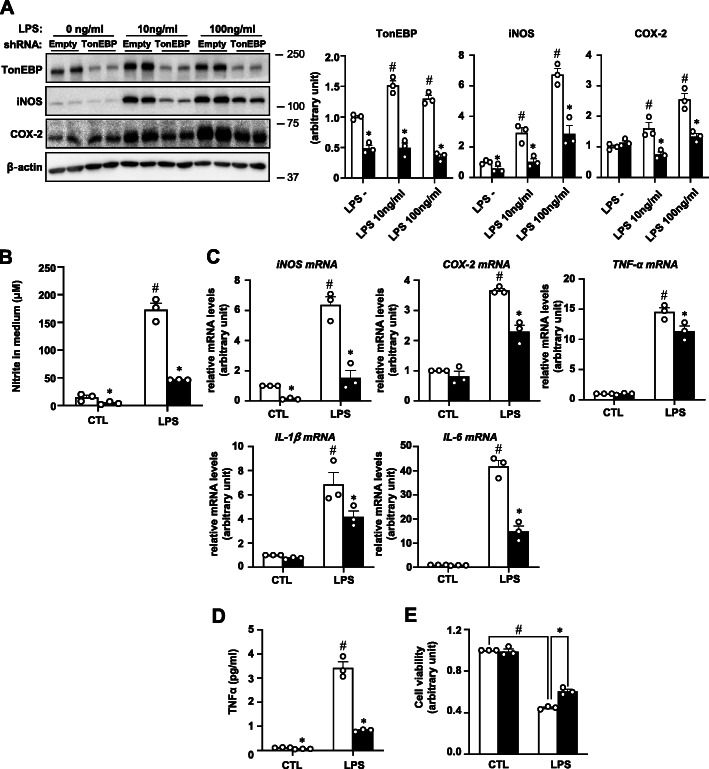
TonEBP in LPS-induced inflammation in BV2 cells. BV2 cells were stably transfected using lentivirus containing empty shRNA (open bars in **b**–**e**) or TonEBP targeting shRNA (solid bars in **b**–**e**) as indicated. **a** The cells were then treated for 24 h with 0 to 100 ng/ml of LPS as indicated and immunoblotted for TonEBP, iNOS, COX-2, and β-actin as shown in left. Right panels show band intensity for TonEBP, iNOS, and COX-2 corrected by intensity of corresponding β-actin band. **b** Nitrite was measured in the media of cells treated for 24 h with 0 (CTL:1x PBS) or 10 ng/ml LPS using Griess reagent. **c** RT-qPCR was performed on cells treated for 1 h (for TNF-α and IL-1β) or 6 h (for iNOS, COX-2, and IL-6) with LPS as in **b**. **d** TNF-α was measured in the medium after the cells were treated for 6 h with LPS. **e** The stably transfected cells were treated for 24 h with 0 (CTL:1x PBS) or 100 ng/ml LPS to obtain conditioned media. HT22 cells grown in regular medium were switched to the conditioned media. After 24 h, cell viability was measured using MTT assay. Mean + SD, *n* = 3. *P < 0.05 compared to empty shRNA. #*P* < 0.05 compared to CTL

In macrophages [11] and hepatocytes [21], TonEBP is a key component of transcriptional machinery for pro-inflammatory gene expression in response to LPS and other signals. TonEBP expression increases dramatically in response to LPS in microglia in association with increased iNOS mRNA expression [13] as in macrophages and hepatocytes. We asked what was the role of TonEBP in LPS-stimulated expression of pro-inflammatory genes in microglia. To answer this question, we first examined a glial cell line BV2 cells. TonEBP expression increased in response to LPS treatment (Fig. 1a) as in microglia discussed above. iNOS expression also increased in association with elevated levels of nitrite which is a degradation product of nitric oxide (Fig. 1b). The increased iNOS expression was associated with elevation of iNOS mRNA levels along with mRNA levels for other pro-inflammatory enzyme and cytokines—COX-2, TNF-α, IL-1β, and IL-6 (Fig. 1c). As expected from the elevated mRNA levels, expression of COX-2 and production of TNF-α also increased (Fig. 1 a and d). When TonEBP was knocked down using lentivirus, mRNA and protein product of the pro-inflammatory enzymes and cytokines all decreased (Fig. 1a–d) indicating a critical role of TonEBP in BV2 cells as in macrophages and hepatocytes.

Activated microglia are known to secrete toxic molecules such as reactive oxygen species including nitric oxide, prostaglandin E_2_, and cytokines that kill neuronal cells [22]. We found that LPS stimulation of microglia produced neurotoxins as measured by reduced survival of a neuronal cell line HT22 cells (Fig. 1e) that might be due to the secretion of neurotoxins by microglia. The production of neurotoxins was reduced in TonEBP-deficient BV2 cells consistent with the reduced production of inflammatory cytokines.

### Cerulenin blocks LPS-induced inflammation by disrupting TonEBP interaction with NF-κB and AP-1

**Fig. 2:**
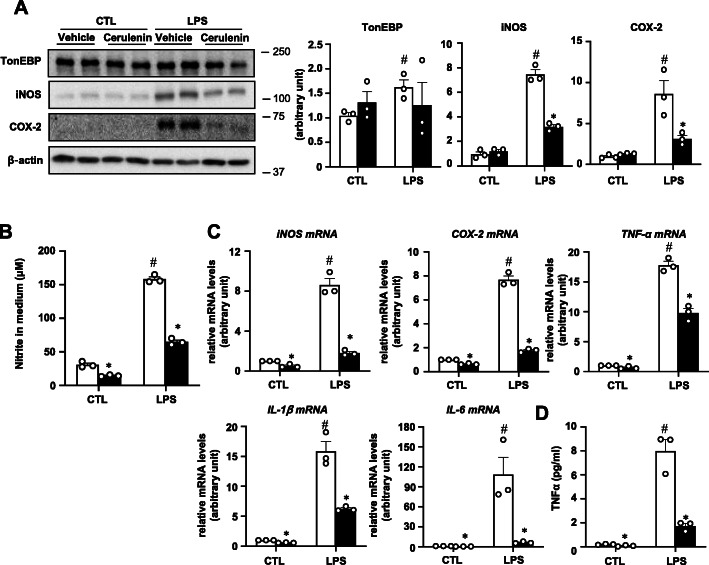
Cerulenin inhibits LPS-induced inflammation in BV2 cells. **a** The cells were pretreated for 1 h with 10 μM cerulenin (solid bars in **b**–**d**) or vehicle (water) (open bars in **b**–**d**), followed by a 24 h treatment with 0 (CTL:1x PBS) or 10 ng/ml of LPS. Immunoblotting and quantitation were performed as in Fig. 1. **b** Nitrite was measured from the media of cells treated as above except that LPS treatment was for 24 h. **c** RT-qPCR was performed on cells treated as in **b** except that LPS was treated for 1 h (for TNF-α and IL-1β) or 6 h (for iNOS, COX-2, and IL-6). **d** TNF-α was measured in the medium after the cells were treated as in **b** for 6 h with LPS. Mean + SD, *n* = 3. *P < 0.05 compared to vehicle. #*P* < 0.05 compared to CTL

In macrophages and hepatocytes, TonEBP is central scaffold in the assembly of NF-κB enhanceosome in which TonEBP connects DNA-bound NF-κB to the histone acetyl transferase p300 [11]. Cerulenin inhibits NF-κB actions by breaking up the assembly of the NF-κB enhanceosome. We used cerulenin to ask whether the NF-κB enhanceosome was responsible for the pro-inflammatory gene expression in BV2 cells. As shown in Fig. 2, cerulenin inhibited mRNA expression and protein expression of the pro-inflammatory enzymes and cytokines demonstrating that the NF-κB enhanceosome was responsible for the transcriptional stimulation. In addition, cerulenin showed clear inhibition on mRNA expression and nitrite production under basal conditions (without LPS treatment).

**Fig. 3:**
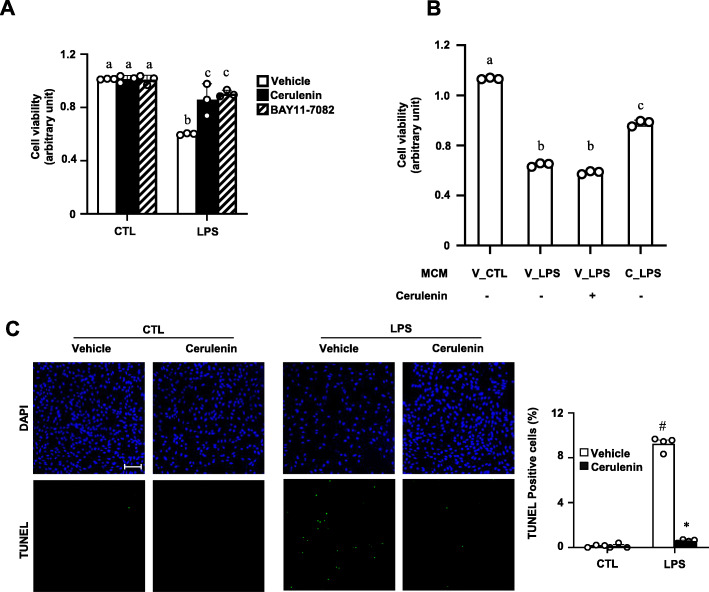
Cerulenin blocks LPS-induced secretion of neurotoxins by BV2 cells. **a** BV2 cells were pretreated for 1 h with vehicle (0.01% DMSO), 10 μM cerulenin, or 5 μM BAY11-7082, followed by treatment for 24 h with 0 (CTL:1x PBS) or 100 ng/ml LPS to obtain conditioned media. HT22 cells grown in regular medium were switched to the conditioned media. After 24 h, cell viability was measured using MTT assay. **b** HT22 cells were cultured in various microglia-conditioned media (MCM) shown with or without addition of 10 μM cerulenin as indicated. Cell viability was measured as above. V, vehicle, C, cerulenin. **c** HT22 cells grown on chamber slides were treated as in **a**: pretreatment with vehicle or cerulenin followed by treatment with CTL or LPS. Fixed cells were analyzed by TUNEL assay. Nuclei were visualized with DAPI. Percent of TUNEL positive cells are shown on the right. Two-way ANOVA with Tukey’s post hoc test was used for multiple comparisons. Bar = 200 μm. Different letters indicate statistical differences at *P* < 0.05. Mean + SD, *n* = 3-4. *P < 0.05, compared to vehicle in **c**. #*P* < 0.05 compared to CTL in **c**

Cell death by the conditioned media was reduced by cerulenin (Fig. 3a) as might be expected from the reduced production of cytokines. In order to exclude the possibility that the presence of cerulenin in the conditioned media was responsible for the reduced neurotoxic activity, cerulenin was directly added to the LPS-conditioned medium (Fig. 3b). The presence of cerulenin did not affect neurotoxic activity indicting that cerulenin in the medium was not responsible for the reduced neurotoxic activity. Since the neurotoxic activity was due to cell death by apoptosis [23], we examined apoptosis in the HT22 cells treated with the conditioned medium. The LPS-conditioned media stimulated apoptosis dramatically (Fig. 3c). On the other hand, conditioned media obtained from BV2 cells treated with LPS in the presence of cerulenin caused much less apoptosis indicating that products of the pro-inflammatory genes were responsible for the neuronal death.

**Fig. 4:**
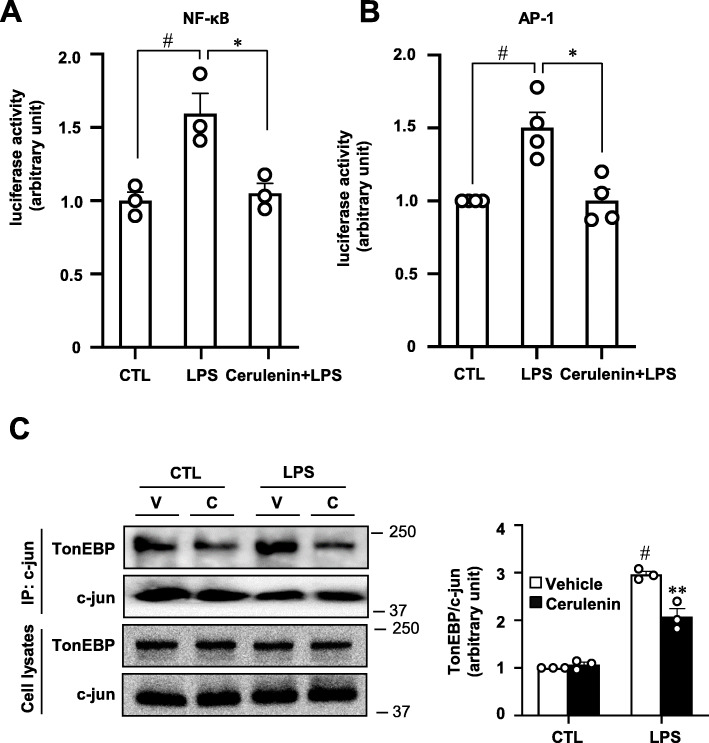
Cerulenin inhibits AP-1 transcriptional activity by disrupting its interaction with TonEBP. **a** BV2 cells were transfected with an NF-κB luciferase reporter. Luciferase activity was measured 3 h after treatment with 100 ng/ml LPS or vehicle (CTL:1x PBS). Where indicated, some cells were pretreated for 1 h with 10 μM cerulenin before treatment with LPS. Mean + SD, *n* = 3. #*P* < 0.05, compared to LPS. *P < 0.05 compared to CTL. **b** BV2 cells were transfected with an AP-1 luciferase reporter. Luciferase activity was measured 3 h after treatment with 100 ng/ml LPS or vehicle (CTL:1x PBS). Where indicated, some cells were pretreated for 1 h with 10 μM cerulenin before treatment with LPS. Mean + SD, *n* = 4. #*P* < 0.05, compared to LPS. *P < 0.05 compared to CTL. **c** MEF cells were pretreated with cerulenin (C) or vehicle (V) followed by treatment with LPS as above. Cell lysates were immunoprecipitated using c-jun antibodies and then immunoblotted for TonEBP and c-jun as shown in left. Right panel shows band intensity for TonEBP corrected by intensity of corresponding c-jun band. **P < 0.05 compared to vehicle. #*P* < 0.05 compared to CTL

We noticed that the production of potential neurotoxins was also inhibited by an NF-κB inhibitor BAY11-7092 as expected (Fig. 3a). Activity of an NF-κB reporter was stimulated by LPS, which was completely blocked by cerulenin (Fig. 4a), consistent with the role of the NF-κB enhanceosome in BV2 cells. Interestingly, we found that an AP-1 reporter was also stimulated by LPS, which was blocked by cerulenin (Fig. 4b). Since AP-1 is activated by LPS and contributes to pro-inflammatory gene expression like NF-κB does [24], we wondered if TonEBP also functioned as a transcriptional cofactor for AP-1. In order to directly test this possibility, we performed co-immunoprecipitation analyses to detect interaction between AP-1 and TonEBP as described previously [11]. When c-jun, a subunit of AP-1, was immunoprecipitated, TonEBP was also brought down (Fig. 4c) demonstrating the interaction between TonEBP and AP-1. Since TonEBP also interacts with p300 in this setting [11], these data provide evidence that TonEBP is a transcriptional cofactor for AP-1. Of note, the interaction was reduced in the presence of cerulenin suggesting that cerulenin disrupted the interaction between TonEBP and AP-1 (Fig. 4c) as it disrupts the interaction between TonEBP and NF-κB [11]. Cerulenin does not change phosphorylation and translocation of NF-κB [11]. Likewise, we found no changes in phosphorylation and nuclear translocation of c-jun (a subunit of AP-1) by treatment of cerulenin (Supplementary Fig. S2) consistent with disruption of the interaction of TonEBP with AP-1 as well as NF-κB. Since there are AP-1 binding sites as well as NF-κB binding sites in the promoter regions of the pro-inflammatory genes [25–27], the TonEBP’s transcriptional cofactor function for both AP-1 and NF-κB is consistent with the cerulenin’s strong inhibition shown in Figs. 2 and 3.

### TonEBP mediates LPS-induced microglial activation

**Fig. 5:**
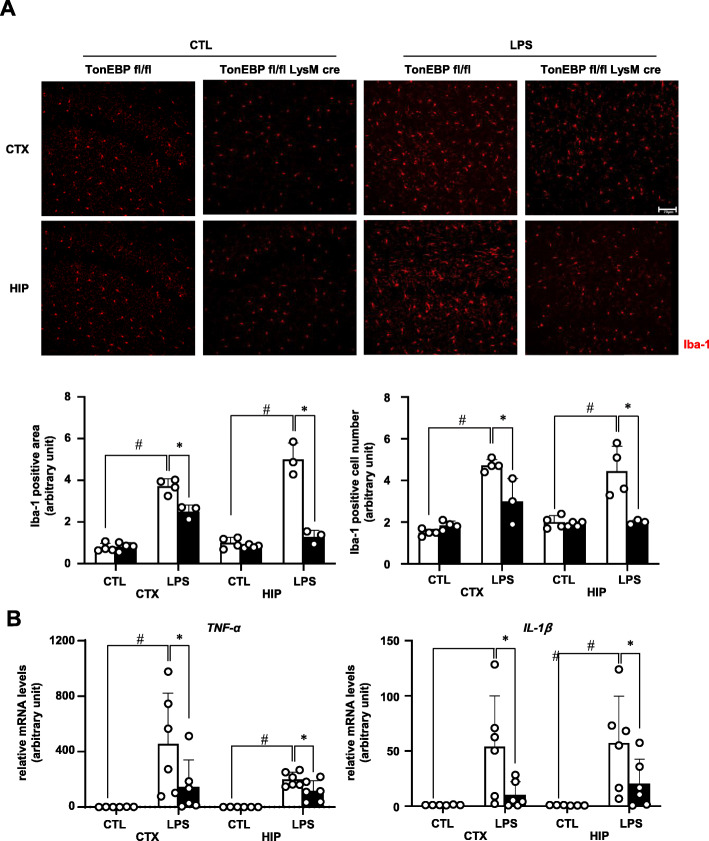
Myeloid TonEBP is required for LPS-induced microglia activation. LPS (3 μg per animal) or vehicle (CTL:1x PBS) was injected into the left ventricle of male TonEBPfl/fl,LysM-cre mice (solid bars) and their TonEBPfl/fl littermates (open bars). Brains were analyzed 3 h later. **a** Brains were perfusion-fixed and immunostained for iba-1. Images from cortex (CTX) and hippocampus (HIP) (top) were analyzed for iba-1 positive areas and iba-1 positive cell numbers (bottom). Bar = 70 μm. **b** RNA was isolated from CTX and HIP, and analyzed by RT-qPCR for TNF-α and IL-1β. Mean + SE, *n* = 3–6. #*P* < 0.05, compared to CTL. *P < 0.05 compared to TonEBP fl/fl

In order to directly assess the role of TonEBP in microglial activation in response to LPS, myeloid-specific TonEBP gene deletion was used. We crossed a floxed TonEBP line (*TonEBP*^*fl/fl*^) with a transgenic line with cre recombinase expression under the control of lysozyme promoter (*LysM-Cre*) to produce *TonEBP*^*fl/fl*^ *LysM-Cre* mice [11]. Efficiency of microglial gene deletion in the *LysM-Cre* line is reported to be 90% [28]. Eight-week-old male *TonEBP*^*fl/fl*^ *LysM-Cre* mice and their *TonEBP*^*fl/fl*^ littermates were used for experiments. To induce inflammation of the brain, we performed intracerebroventricular injection of LPS as described [13]. Cortex (CTX) and hippocampus (HIP) were processed for immunostaining and RNA analysis 3 h after LPS injection. Microglial activation was detected by immunohistochemical visualization of Iba-1, a marker for activated microglia [29]. Based on increased area of Iba-1-positive area and number of Iba-1-positive cells, LPS injection resulted in a clear activation of microglia in the *TonEBP*^*fl/fl*^ animals (Fig. 5a). The activation was associated with elevated levels of mRNA for TNF-α and IL-1β (Fig. 5b). Both the microglial activation and elevation of mRNA levels were blocked in the animals with myeloid-specific TonEBP deletion, i.e., in the *TonEBP*^*fl/fl*^ *LysM-Cre* animals. These data demonstrate that myeloid TonEBP is required for inflammatory microglial activation.

**Fig. 6:**
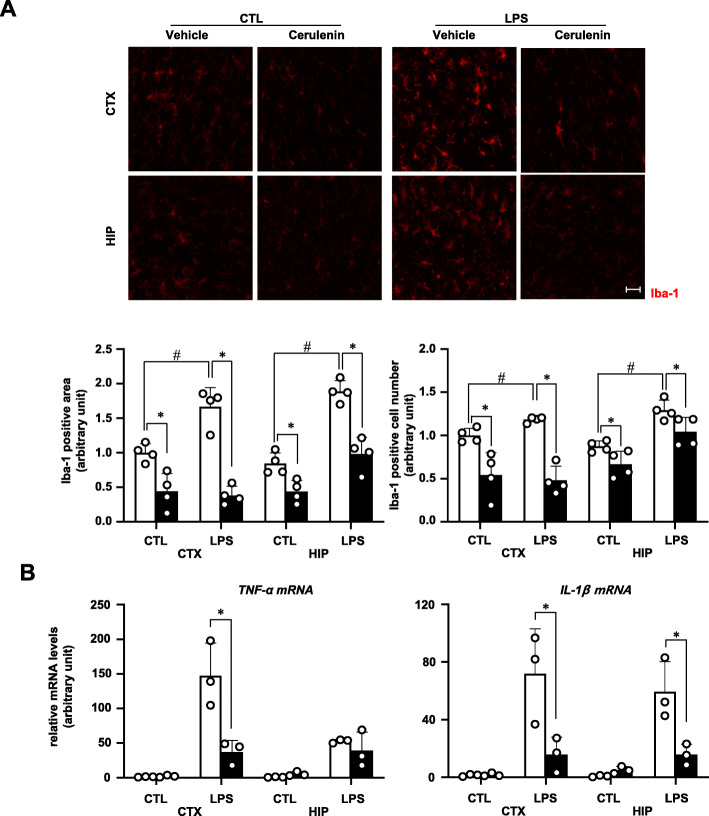
Cerulenin inhibits LPS-induced microglia activation. Cerulenin (6 nmol per animal, solid bars) or vehicle (water) (open bars) was injected into the left ventricle. One hour later, LPS (3 μg per animal) or vehicle (CTL:1x PBS) was injected again. Brains were analyzed 3 h later. **a** Brains were perfusion-fixed and immunostained for iba-1. Images from cortex (CTX) and hippocampus (HIP) (top) were analyzed for iba-1 positive area and iba-1 positive cell number (bottom). Bar = 70 μm. **b** RNA was isolated from CTX and HIP and analyzed by RT-qPCR for TNF-α and IL-1β. Mean + SE, *n* = 3–4. #*P* < 0.05, compared to CTL. #*P* < 0.05, compared to CTL. *P < 0.05 compared to vehicle

Next, we asked whether TonEBP-dependent actions of NF-κB and AP-1 were involved in the microglial activation. Cerulenin was injected into the brain 1 h prior to LPS injection described above. Cerulenin blocked the activation of microglia (Fig. 6a) and the increased expression of mRNA for TNF-α and IL-1β (Fig. 6b). These data show that TonEBP mediates microglial activation by stimulating the transcriptional activity of NF-κB and AP-1.

### TonEBP mediates LPS-induced memory loss in association with reduced number of neuronal cells

**Fig. 7:**
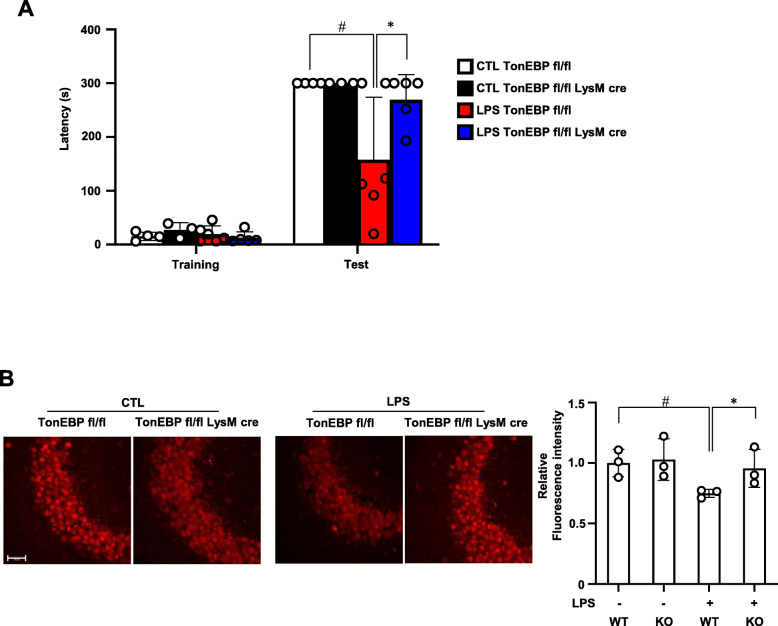
Myeloid TonEBP is required for blocking LPS-induced loss of short-term memory. **a** LPS (3 μg per animal) or vehicle (CTL:1x PBS) was injected into the left ventricle of male TonEBPfl/fl,LysM-cre mice (KO in **b**) and their TonEBPfl/fl littermates (WT in **b**). Two weeks later, passive avoidance test was performed followed by perfusion fixation of the brains. Latency in seconds in passive avoidance test. Mean + SE, *n* = 3–6. *P < 0.05 B NeuN immunostaining in CA3 region of hippocampus. Florescence intensity of the immunostaining was shown on the right. Bar = 50 μm. Mean + SE, *n* = 3. #*P* < 0.05, compared to CTL. *P < 0.05 compared to TonEBP fl/fl

Since TonEBP mediates LPS-induced neuroinflammation (see above) and neuroinflammation induces memory loss [30, 31], we asked whether TonEBP is required from LPS-induced memory loss. The male *TonEBP*^*fl/fl*^ *LysM-Cre* mice and their *TonEBP*^*fl/fl*^ littermates were injected with LPS or vehicle as described above. Two weeks later, passive avoidance test was performed to assess short-term memory followed by histological analysis of the brains. Short-term memory measured by latency in passive avoidance test was halved in the *TonEBP*^*fl/fl*^ mice (Fig. 7a) in association with reduced number of neurons in the CA3 region of the hippocampus (Fig. 7b). The reduced neuronal cell number was likely due to cell death as observed in BV2 cells whose TonEBP was knockdown (Fig. 1e). These changes were not observed in the *TonEBP*^*fl/fl*^ *LysM-Cre* mice suggesting that TonEBP is required for the LPS-induced neuronal cell death and memory loss.

**Fig. 8:**
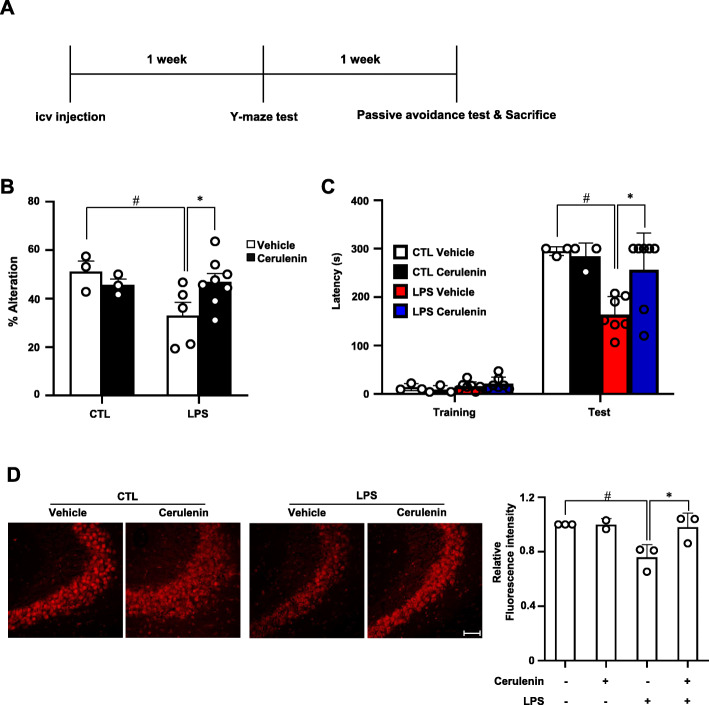
Cerulenin blocks LPS-induced loss of spatial and short-term memory. **a** Experimental scheme. Cerulenin (6 nmol per animal) or vehicle (water) was injected into the left ventricle. One hour later, LPS (3 μg per animal) or vehicle (CTL:1x PBS) was injected again. One week later, Y-maze test was performed to assess spatial memory. Two weeks later, passive avoidance test was performed followed by perfusion fixation of the brains. **b** Percent alternations in Y-maze test. **c** Latency in seconds in passive avoidance test. **d** NeuN immunostaining in CA3 region of hippocampus. Florescence intensity of the immunostaining was shown on the right. Bar = 50 μm. Mean + SE, *n* = 3–8. #*P* < 0.05, compared to CTL. *P < 0.05 compared to vehicle

In order to examine the role of TonEBP-mediated actions of NF-κB and AP-1, we examined the effects of cerulenin. LPS or vehicle was injected into the brain of 8-week male mice as described above. Y-maze test was performed after 1 week to assess spatial memory, and passive avoidance test after 2 weeks as described above (Fig. 8a). In animals injected with LPS, both spatial memory and short-term memory were significantly reduced (Fig. 8b, c) in association with reduced number of neurons in the CA3 region of the hippocampus (Fig. 8d). These changes were blocked when cerulenin was pretreated 1 h before the LPS administration suggesting that TonEBP-mediated neuroinflammation causes neuronal cell death and memory loss. (Supplementary Fig. 1)

## Discussion

The activation of microglia contributes to aging [32, 33] and the pathogenesis of neurodegenerative diseases such as Alzheimer’s disease [34–36]. NF-κB and AP-1 are essential transcriptional regulators for the activation of microglia [37–39] as well as for pro-inflammatory activation of macrophages [40, 41]. In macrophages, TonEBP is a key mediator of LPS-induced activation of pro-inflammatory gene expression [11, 42, 43]. This is achieved in two ways: elevation of TonEBP expression and TonEBP functioning as transcriptional cofactor for NF-κB. Here, we find that TonEBP has the same role in microglia. In addition, we discover that TonEBP is also the transcriptional cofactor for AP-1. In settings of LPS-induced microglial activation and neuronal damage, the TonEBP-mediated actions of AP-1 and NF-κB are required for the inflammatory activation of microglia based on observations from TonEBP deleted animals and effects of cerulenin, which inhibits AP-1 and NF-κB by blocking their interactions with TonEBP and p300. Thus, TonEBP mediates the LPS-induced microglial activation and neuronal damage as transcriptional cofactor for AP-1 and NF-κB, which are the major transcription factors in the pro-inflammatory gene expression.

Neuroinflammation is associated with aging [44, 45], metabolic diseases [46], and a variety of neurodegenerative diseases such as Alzheimer’s disease [47, 48], Parkinson’s disease [49], multiple sclerosis [50], and amyotrophic lateral sclerosis [51]. Various causes including obesity, diabetes, hypertension, and even lifestyles increase systemic inflammatory response [52, 53]. Systemic increase in pro-inflammatory mediators (cytokines, chemokines, ROS, and secondary messengers) enhances microglial cell activation [53], which makes neuroinflammation as a disease-promoting factor in neurodegenerative diseases. The activated microglia cause neuronal death via inflammatory factors, A1 astrocyte activation, and microglia-mediated synapse loss [5, 6]. Our data presented here uncovers the role of microglial TonEBP in neuronal death. Targeting the TonEBP/AP-1/NF-κB pathway should be an attractive strategy to prevent the neuronal death. In this regard, the actions of cerulenin in inhibiting the prototypical pro-inflammatory transcription factors AP-1 and NF-κB by disrupting their interactions with TonEBP rather than direct inhibition (such as nuclear translocation or phosphorylation) provide a new mode of intervention and potential therapeutics.

## Conclusions

In sum, this study has shown that TonEBP is required for the LPS-induced microglial activation and pro-inflammatory gene expression. TonEBP mediates the transcriptional activity of the classical pro-inflammatory transcription factors NF-κB and AP-1. Deletion of TonEBP in myeloid cells or treatment with cerulenin blocks the TonEBP-mediated activation of NF-κB and AP-1, microglial activation, and subsequent neuronal damages. Thus, we have delineated intracellular pathways involved in the microglial activation relevant to neuronal damage.

## Supplementary Information

**Additional file 1: Figure S1**. Model of TonEBP in microglia-mediated memory loss induced by LPS. **Figure S2**. Effects of cerulenin in phosphorylation and nuclear translocation of c-jun. **Table S1**. Primer sequences for quantitative PCR.

## Data Availability

The datasets and materials used and/or analyzed during current study are available from the corresponding author on reasonable request.

## Abbreviations

ROS: Reactive oxygen species
CNS: Central nervous system
TonEBP: Tonicity-responsive enhancer binding protein
NFAT5: Nuclear factor of activated T cells 5
NF-κB: Nuclear factor-κB
TNF-α: Tumor necrosis factor-α
COX-2: Cyclooxygenase-2
LPS: Lipopolysaccharide
DMEM: Dulbecco’s Modified Eagle’s Medium
FBS: Fetal bovine serum
MEFs: Mouse embryonic fibroblasts
PBS: Phosphate-buffered saline
MTT: 3-(4,5-dimethylthiazol-2-yl)-2,5-diphenyltetrazolium bromide
CTX: Cortex
HIP: Hippocampus
