# Supplementary material for: Microglial TonEBP mediates LPS-induced inflammation and memory loss as transcriptional cofactor for NF-κB and AP-1
Source: J Neuroinflammation. 2020 Dec 8;17:372. doi: 10.1186/s12974-020-02007-9 (PMC7722447; doi:10.1186/s12974-020-02007-9)
Supplement: Supplementary file 1 — Additional file 1: Figure S1. Model of TonEBP in microglia-mediated memory loss induced by LPS. Figure S2. Effects of cerulenin in phosphorylation and nuclear translocation of c-jun. Table S1. Primer sequences for quantitative PCR. [file 12974_2020_2007_MOESM1_ESM.docx]

**Supplemental Information**

**Microglial TonEBP mediates LPS-induced inflammation and memory loss as transcriptional cofactor for NF-κB and AP-1**

Gyu Won Jeong, Hwan Hee Lee, Whaseon Lee-Kwon, Hyug Moo Kwon^*^

**Supplemental Figure Legends**

**
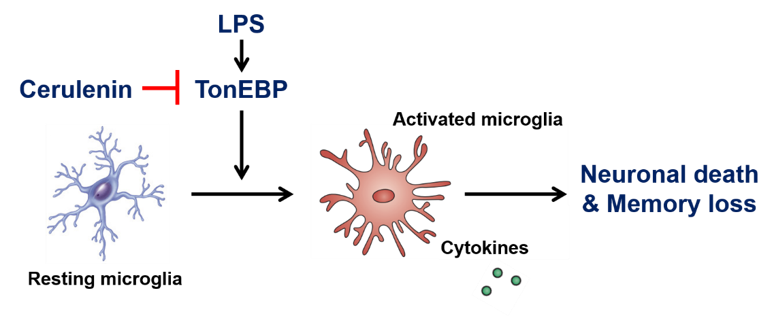
**

**Supplementary Figure 1. Model of TonEBP in microglia-mediated memory loss induced by LPS.**

**
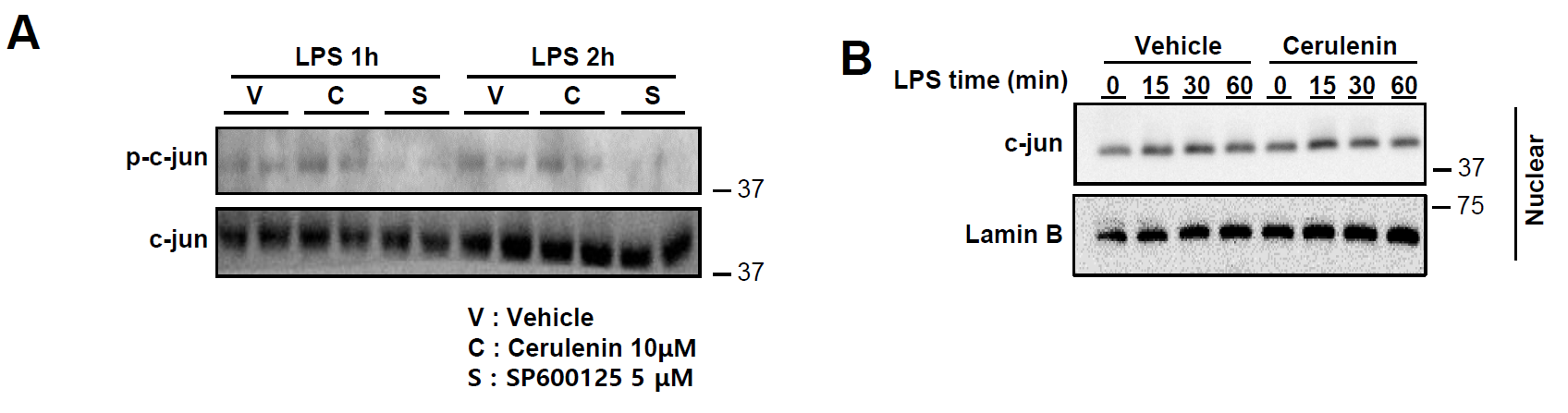
**

**Supplementary Figure 2. Effects of cerulenin in phosphorylation and nuclear translocation of c-jun.**

A BV2 cells were pretreated for 1 h with vehicle (V), 10 μM cerulenin (C), or 5 μM SP600125 (S, a JNK inhibitor), followed by treatment with 10 ng/ml of LPS for 1 or 2 h as indicated. Cell lysates were immunoblotted for phosphorylated c-jun (p-c-jun) and c-jun. B Cells were pretreated for 1 h with vehicle or cerulenin, followed by a treatment with LPS for up to 60 min as indicated. Nuclear fractions were obtained as described in Methods and immunoblotted for c-jun and Lamin B.

**Supplementary Table 1. Primer sequences for quantitative PCR.**

**
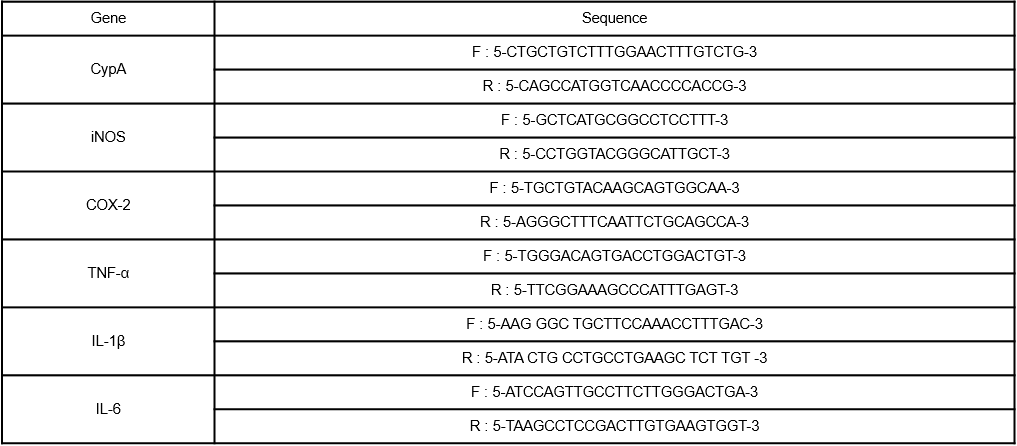
**
